# Purification of Coffee Polyphenols Extracted from Coffee Pulps (*Coffee arabica* L.) Using Aqueous Two-Phase System

**DOI:** 10.3390/molecules28155922

**Published:** 2023-08-07

**Authors:** Phuong Hong Le, Linh Thuy Thi Ho, Dao Hong Thi Le, Viet Nguyen

**Affiliations:** Department of Chemical Engineering, Faculty of Chemical Engineering and Food Technology, Nong Lam University, Ho Chi Minh City 70000, Vietnam; linhthuy41100@gmail.com (L.T.T.H.); hongdaole21102@gmail.com (D.H.T.L.); nbviet@hcmuaf.edu.vn (V.N.)

**Keywords:** aqueous two-phase system, antioxidant activity, coffee pulp extract, polyphenols, purification

## Abstract

Coffee pulp is an abundant residue from the coffee industry, but it still contains large amounts of valuable compounds such as polyphenols. The extraction of polyphenols from coffee pulp by the conventional method is accompanied by contaminated compounds. This study, therefore, applied an aqueous two-phase system consisting of different ratios of ethanol/ammonium sulfate to eliminate impurities from coffee-pulp crude extract. The purification efficiency was evaluated via total polyphenol content, antioxidant activity and two major polyphenols in coffee pulps including chlorogenic acid and caffeic acid. Results showed that phenolic compounds mostly predominated in the alcohol-rich phase in which the antioxidant activity was greatly increased after the purification process. Compared to un-purified crude-coffee extract, the antioxidant activity of the purified samples increased approximately 34%, which was assumed to occur due to the slight increase of chlorogenic acid and caffeic acid. Fourier-transform infrared spectroscopy supported the effectiveness of the purification process by eliminating some impurities.

## 1. Introduction

Coffee pulp is generally considered to be a waste product that represents approximately 29% of the dry weight of the coffee cherry [1]. However, this agricultural by-product still contains high value-added compounds such as carbohydrate, pectin, cellulose, hemicellulose, and bioactive compounds [2,3]. The recovery of such value-added compounds brings not only economic benefits but also helps to reduce environmental threats. Coffee pulp contains an appreciable amount of phenolic and flavonoid compounds, which have attracted great interest because of their anti-inflammatory, anti-microbial, anti-allergic, and anti-carcinogenic properties [1]. Maceration methods applied to extract natural compounds from plant materials utilize high amounts of solvents, obtaining not only polyphenols but also other compounds that require other purification treatments for further utilization [4].

The aqueous two-phase system (ATPS) is a liquid–liquid fractional technique attracting great interest because of its potential for the extraction, separation, and purification of chemical compounds [5]. The separation of the compounds is mainly due to their different solubility in different aqueous solutions [6]. ATPS can be performed by mixing two incompatible solvent or solution, including polymer/polymer, polymer/salt, ionic liquid/salt, or alcohol/salt solutions, that cause partitioning into two distinct phases [7]. Compared to other purification methods such as high-peformance liquid chromatography or ultra-filtration, ATPS has more advantages, including low operational cost, low energy consumption, procedural simplicity, and ease of scaling up the process [8,9,10].

In the ATPS process, a combination of alcohol (ethanol (EtOH), methanol (MeOH) and propanol) and salt (i.e., potassium hydrophosphate, K_2_HPO_4_; sodium dihydrogen phosphate, NaH_2_PO_4_; disodium hydrogen phosphate, NaH_2_PO_4_ or ammonium sulphate, (NH_4_)_2_SO_4_) is potentially applied to purify the bioactive compounds due to the ease of preparation and non-toxic solvents. Inorganic salts including K_2_HPO_4_, (NH_4_)_2_SO_4_, Na_2_CO_3_ were reported to be effective for an easy, stable, and transparent solution formation [11]. By using the EtOH/ phase system, some studies were successful in removing nitrogen-containing compounds in anthocyanin extract from *Peristrophe bivalvis* (L.) *Merr.* leaf (Acanthaceae) [6] to eliminate cancerogenic ethyl carbamate from red wine [12], to purify phenolic compounds in berry juices [13] and roselle calyces (*Hibiscus sabdariffa*) [14], or to extract edible polysaccharides from medicinal plants [13]. Some of the applications of ATPS using EtOH/inorganic salt are listed in Table 1.

Most studies have focused on comparing the select suitable types of APTS to carry out the extraction of bioactive compounds from plant materials. Moreover, the affecting factors such as temperature, time, and pH have also been studied to optimize the extraction yield [4,21,22]. However, there are no studies evaluating the efficiency of ATPS on the purification of natural compounds extracted from fruit peels, especially in the case of polyphenol extracted from coffee pulps. Furthermore, the differences in the chemical properties of the purified and unpurified samples such as total polyphenol content (TPC) and the antioxidant activity (AA) of the extract have also not been investigated.

The objective of this study was to focus on extracting and purifying coffee-pulp polyphenols. Three different ATPSs of EtOH and (NH_4_)_2_SO_4_ were applied to purify coffee-pulp crude extract, and the effectiveness of the purification procedure was evaluated based on polyphenol content and the antioxidant capacity of the product.

## 2. Results and Discussion

### 2.1. Crude Extract of Coffee Pulp

**Figure 1:**
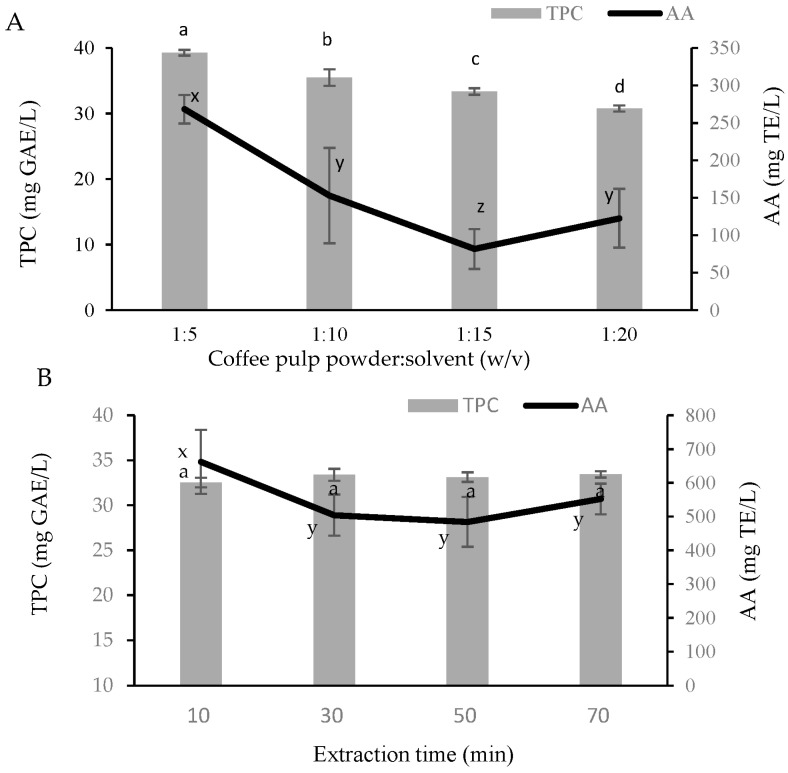
Effects of ratio of material:solvent (**A**) and extraction time (**B**) on the TPC and AA of the coffee pulp extract. Superscript letters (a, b, c, d) and (x, y, z) expressed the significant difference (*p* < 0.05) in TPC and AA, respectively.

Single-factor experiments, including the ratio of material:solvent and soaking time, were performed to study the effect of the extraction conditions on the quality of phenolic compounds extracted from coffee pulps. Figure 1 shows the first significantly affected TPC and AA of the extract (*p* < 0.05). The TPC value varied from 30.75 to 39.26 mg GAE/L, while the AA changed from 81.67 to 286.33 mg TE/L. The extract obtained the highest TPC and AA at a ratio of 1:5 (*w*/*v*). When the amount of solvent was increased, the TPC of the extract was slightly decreased, whereas AA was greatly decreased, which could have been due to the dilution effect. However, prolonging the extraction time did not significantly affect the TPC and AA of the product. With the assistance of an ultrasonic wave, most polyphenols in coffee pulps were quickly released after 10 min of extraction. Phuong et al. [23] also reported that the TPC of fruit peels was unchanged within the extraction time (20–40 min). Furthermore, the exposure of the extract to the environment could result in decreasing its AA due to oxidation.

### 2.2. Phase Ratio (R), Partition Coefficient (K) and Purification Efficiency (E)

**Figure 2:**
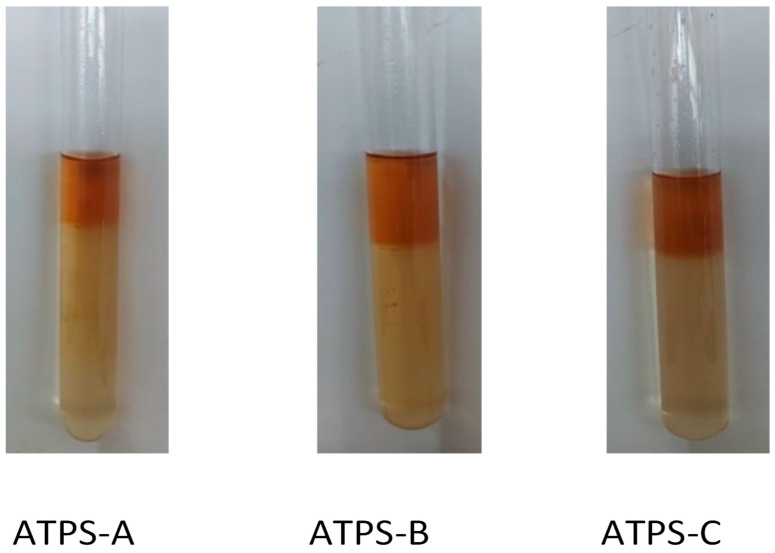
Phase separation of coffee-pulp extract in three aqueous two-phase systems.

Three ATPSs (ATPS-A, ATPS-B and ATPS-C) with a difference in ratio of EtOH/(NH_4_)_2_SO_4_ and water (Table 1) were used to purify polyphenol in the coffee pulp extract. It could be seen that the volume of each phase in each ATPS was clearly different and differentiated via color (Figure 2). Phase ratio represents the changing volume of the two phases after purification and was calculated by the ratio of the volume of the alcohol-rich phase and salt-rich phase, ranging from 0.33 to 0.47, showing less volume of the top phase compared to the volume of the bottom phase (Table 2). A previous study has reported that the solubility of salt and the phase ratio of the top and bottom phase volumes can directly affect the operating feasibility of ATPS [24]. This was due to the solubility and polarity properties; the polyphenols were mostly distributed in the alcohol-rich phase of the EtOH/(NH_4_)_2_SO_4_ system, which was clearly expressed via phase color and the partition coefficient [13]. As hypothesized, the partition coefficients of the three ATPSs were greatly higher than 1, meaning that most of phenolic compounds predominated in the alcohol phase [25]. The partition coefficients of TPC obtained in this study ranged from 17.82–22.10%. In fact, ATPS using EtOH/(NH_4_)_2_SO_4_ was reported as obtaining the highest partition coefficient among EtOH/NaH_2_PO_4_, propanol/glucose, or propanol/maltose [7]. Furthermore, the purification efficiency of TPC in this study was in the range from 88.01–90.35%. These results are in line with the findings of a previous study, which reported that the extraction efficiency of polyphenols from ramie leaves using EtOH/salt ATPS greatly fluctuated from 18.86 to 93.58%, depending on the phase system [22].

In our study, EtOH concentration ranged from 26% to 32% (*w*/*w*), and (NH_4_)_2_SO_4_ concentration was from 16–20% (*w*/*w*). Zhang et al. [4] also reported these ranges were the tentatively suitable concentration for extraction and purification of phloridzin from *Malus Micromalus* Makino with the EtOH/(NH_4_)_2_SO_4_ system. Among the three ATPS systems, ATPS-B had the lowest (NH_4_)_2_SO_4_ concentration and the highest EtOH concentration, resulting in the highest volume ratio and the lowest partition coefficient. However, no difference in purification efficiency was observed in the three systems.

### 2.3. Total Polyphenol Content, Antioxidant Activity and Characterized Polyphenols in the Purified Coffee Pulp Extract

The chemical properties of the unpurified and purified coffee extract are presented in Table 3. The TPC of the coffee pulp extract was 37.54 mg GAE/L, which was slightly increased after the purification process, but there was no difference between purified and unpurified samples (*p* > 0.05). Interestingly, the AA of the purified samples was significantly increased (*p* < 0.05), attaining 778.05 ± 21.11 mg TE/L. After purifying, the AA of coffee extract increased approximately 34%, but there were also no differences among the three systems. These results show that the AA has a correlation with TPC, as higher TPC leads to higher AA. However, there was no study to evaluate the AA of purified samples to compare with our obtained results.

In fact, the highest chlorogenic acid (CGA) and caffeic acid (CFA) concentrations in coffee extract after purifying were 149.63 ± 12.79 and 233.12 ± 24.80 mmol/L, respectively, compared to 138.59 ± 7.77 and 218.66 ± 19.94 mmol/L of the unpurified samples. Also, no differences in the content of these polyphenols were obtained from the three ATPSs (*p* > 0.05). However, results obtained in our study were different from those reported by Chong et al. [7], who used similar phase systems to purify bioactive compounds from haskap leaves. In this study, the maximum CGA and TPC yields were obtained when the ratio of EtOH/(NH_4_)_2_SO_4_ was 32/16 and 28/20, respectively, equivalent to ATPS-B and ATPS-C in our study. When these phases were applied on coffee pulp extract, the contents of TPC and CGA purified by using ATPS-A and ATPS-C were not significantly different. The differences in the purifying effectiveness of the system were attributed to the salting-out effect produced by increasing the salt concentration in the bottom phase and the properties of the compound that we wanted to remove from the extract [26].

CGA and CFA are the major polyphenols present in coffee pulp [1,27]. In the literature review, polyphenols extracted from plants or herbs were found to migrate to the top phase of ATPS formed by EtOH/(NH_4_)_2_SO_4_. Chong et al. [7] also revealed that the yield of CGA from ATPS with EtOH/(NH_4_)_2_SO_4_ was higher than that of the conventional extract and phase system using propanol/glucose or propanol/maltose. In another study, the authors reported that CGA has a very high AA, even higher than gallic acid [28]. This result supported the high AA of the purified coffee extract compared to unpurified samples. Several studies reported the efficiency of ATPS when purifying the plant extract by removing sugar [9] or nitrogen compounds [6], but the efficiency was different depending on the phase system and natural compounds. A previous study was conducted on the extraction of polyphenols by using different ATPSs, showing that the EtOH/salt system was better than propanol/sugar, giving higher TPC and flavonoids yields of the extract [7]. Thus, removing the impurites by using ATPS could result in increasing the quality of the purified extract and improving samples of AA.

### 2.4. FTIR Spectrum of Polyphenol Extracts before and after Purification

**Figure 3:**
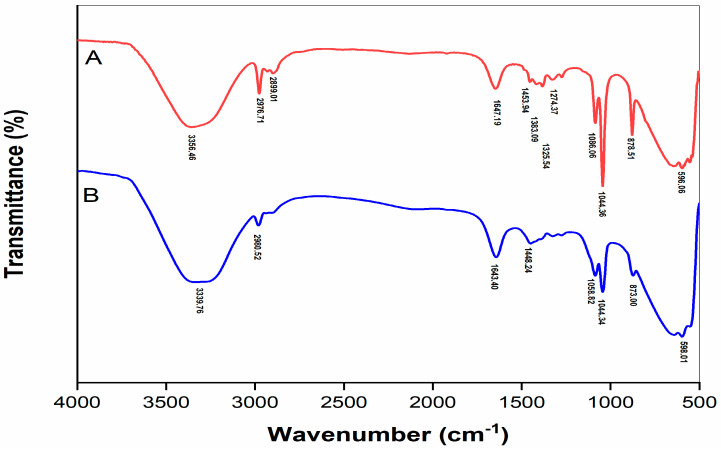
Fourier transform infrared (FTIR) spectrum of coffee-pulp crude extract before (A) and after purification (B).

The Fourier transform infrared (FTIR) spectrum of the purified and un-purified coffee extract is depicted in Figure 3. The region of 3400–3200 cm^−1^ indicates a symmetric and asymmetric stretching of the hydroxyl group (O-H), H-bonding stretching, which is characteristic of phenolic compounds. The major absorption band around 3300 cm^−1^ may be associated with the stretching of O-H and stretching vibration of C-H. The peaks between 596 and 1647 cm^−1^, the fingerprint zone, could be attributed to C=C aromatic ring stretching (1643, 1647 cm^−1^) and several aromatics out-of-plane C-H (873, 878 cm^−1^) and in plane (1044 cm^−1^) [29]. In comparison with the unpurified sample, the purified extract displayed the absence of peaks at 1453.94 cm^−1^, 1383.09 cm^−1^, 1325 cm^−1^ and 1274.37 cm^−1^, which may be related to the OH groups of lignin and polysaccharides [30]. According to the report of Cordero and Marquez [31], peaks characterized for the structure of cellulose, hemicellulose, and lignin could be fluctuated in a certain range. These peaks close to the peaks of 1450 cm^−1^ and 1375 cm^−1^ of the unpurified coffee extract which were reported as stretching of the C-H bond of the acetyl group presence in the lignin and angular deformation of C-H groups of cellulose, respectively [32,33], while the peak at 1325.25 cm^−1^ is attributed to the C-O stretch of lignin [31]. Therefore, the FTIR result could support the differences in chemical composition of the purified and unpurified coffee-pulp extract.

## 3. Materials and Methods

### 3.1. Chemicals and Materials

The coffee pulps (*Coffee arabica* L.) were obtained from Lam Dong province, Vietnam. Folin–Ciocalteu reagent, 2,2′-azinobis (3-ethylbenzothiazoline-6-sulfonic acid) radical cation (ABTS^●+^), chlorogenic acid (CGA), and caffeic acid (CFA) were supplied by Sigma-Aldrich, Singapore; gallic acid was purchased from Himedia, India. All other chemicals were of analytical grade.

### 3.2. Preparation of the Crude Extract

First, experiments were conducted to determine the extraction conditions of polyphenols from coffee pulps. Based on the preliminary test, dried coffee-pulp powder (moisture content of 13.9%) was mixed with EtOH 75% in a ratio of 1:5, 1:10, 1:15 and 1:20 (*w*/*v*) for different time (10–70 min). The extraction was carried out at 30 °C in an ultrasonic bath with a power of 40 KHz. The extract was then centrifuged at 4000 rpm at 25 °C for 15 min to obtain the supernatant, which was evaluated for the TPC and AA. The extraction condition obtaining the extract having the highest TPC and AA was applied to extract bioactive constituents from coffee pulp that was used for further purification steps.

### 3.3. Phase Diagram Building and ATPS Preparation

The phase diagram of ATPS is a binodal curve formed by a series of equilibrium points between the EtOH and (NH_4_)_2_SO_4_ solution. It was built by mixing a stock solution of EtOH (60–100%) with (NH_4_)_2_SO_4_ solution (1–40%) at different ratios. Briefly, a known volume and concentration of (NH_4_)_2_SO_4_ solution was titrated with EtOH, for which the equilibrium points were determined when the mixtures turned turbid [6].

Similar to the previous study of Chong et al. (2020), three ATPS above the equilibrium line were applied to crude extracted from coffee pulps. Details of the phase-forming components of each phase system, called ATPS-A, ATPS-B and ATPS-C, are supplied in Table 4. Briefly, an amount of (NH_4_)_2_SO_4_ was weighted in a falcon tube, and then water was added and thoroughly vortexed to dissolve (NH_4_)_2_SO_4_. After that, EtOH was added and shaken well before an aliquot of crude coffee-pulp extract was added and mixed well by using a vortex. Due to the incompatibility of an alcohol and salt solution, the phase separation could be achieved in few minutes. The mixture was kept stable in the dark at room temperature for 10 min. Two phases were completely formed with different colors (Figure 2). The top phase was alcohol, while the bottom phase was salt solution, which was then separated by using a micropipette.

### 3.4. Analysis Methods

#### 3.4.1. Determination of Volume ratio (*R*), Partition Coefficient (*K*) and Purification Efficiency (*E*)

The volumes of the alcohol-rich phase (*V_t_*) and the salt-rich phase (*V_b_*) were separated and recorded. Volume ratio (*R*), partition coefficient (*K*) and purification efficiency (*E*) were determined from Equations (1)–(3), respectively.

(1) $$R=\frac{V_{t}}{V_{b}}$$

(2) $$K=\frac{C_{t}}{C_{b}}$$

(3) $$E\;\left(\%\right)=\frac{K}{K+\frac{1}{R}}\times 100\%$$

where *C_t_* and *C_b_* are the total phenolic content (TPC) in the alcohol-rich phase and salt-rich phase, respectively.

*V_t_* and *V_b_* are the volumes of the alcohol-rich phase and salt-rich phase, respectively.

#### 3.4.2. Total Phenolic Content

TPC was determined by using Folin–Ciocalteu’s phenol reagent (FC) [23]. First, 1 mL extract was mixed with 0.5 mL of FC 10% and kept for 6 min at room temperature. After that, 1.5 mL of sodium carbonate (7.5%, *w*/*v*) was added to the mixture, mixed well, and kept for 2 h in the dark at room temperature. The absorbance was measured at 760 nm (UV11, MRC-Lab, Harlow, Essex, UK). The concentration of total phenolic compounds was calculated based on the external standard curve of gallic acid and expressed as mg of gallic acid equivalent (GAE) per L.

#### 3.4.3. Antioxidant Activity Determination

ABTS assay: The analysis procedure followed the method as described by Phuong et al. [23]. First, the ABTS^●+^ stock solution was prepared by mixing 7.0 mM ABTS^●+^ solution and 2.45 mM potassium persulfate solution (1:1, *v*/*v*) and kept for 16 h in the dark at room temperature. An ABTS^●+^ working solution was freshly prepared by diluting the ABTS^●+^ stock solution with 90% MeOH to obtain an absorbance of 0.7 ± 0.02 at 734 nm using a spectrophotometer. In a test tube, 20 μL of the extract was allowed to react with 2 mL of ABTS^●+^ working solution for 5 min in the dark at room temperature. The absorbance was spectrophotometrically measured at 734 nm and the results were expressed in g of Trolox equivalent (TE) per L.

#### 3.4.4. Analysis of CFA and CGA

CGA and CFA in the alcohol-rich phase were spectrophotometrically determined with some modifications [34]. Briefly, an aliquot of the extract (50 μL) was mixed with 4 mL of acetic acid 1%; then, 4 mL of acetonitrile was added and thoroughly mixed. After that, 0.5 g of NaCl and 1 g of MgSO4 were added to the mixture, shaken well and centrifuged at 4 °C at 4000 rpm for 15 min. The top phase was obtained to spectrophotometrically measure the absorbance of CFA and CGA at 320 and 325 nm, respectively. CFA and CGA (Sigma 99.9%) were used to prepare standard curves, and the results were expressed as mol/L.

#### 3.4.5. Fourier-Transform Infrared Spectroscopy (FTIR)

The infrared spectrum (with a wavelength in a range of 4000–400 cm^−1^) of the coffee extract and purified sample was determined using a FTIR spectrophotometer (Bruker Tensor 27, Billerica, MA, USA).

### 3.5. Statistical Analysis

Data were presented as mean ± standard deviation. The effect of ATPS on the purification of polyphenol in the coffee extract was evaluated by ANOVA and Tukey at 95% confidence interval using SPSS software (v.20, IBM, New York, NY, USA).

## 4. Conclusions

Plant material extracts often contain phenolic and other compounds in their mixture which may limit their use in foods. Purification, therefore, is a necessary step to remove the unwanted compounds and concentrate the wanted compounds, but this process usually requires energy, processing time, equipment, and chemical and economic issues. The aqueous two-phase system is a liquid–liquid system and has been successfully used in the laboratory to separate bioactive compounds from several plant extracts. Our study, therefore, applied the aqueous two-phase system consisting of ethanol and ammonium sulfate to purify polyphenol extracted from coffee pulp. Although the investigated ATPS conditions did not contribute to significantly increasing the content of phenolic compounds (such as chlorogenic acid and caffeic acid) in coffee extract, the antioxidant activity of the extract greatly increased up to 34% after the purification process. Moreover, the aqueous two-phase system was effective in removing unwanted compounds (such as lignin and carbohydrates) from coffee-pulp extract, expressed via the Fourrier-transform infrared spectroscopy of two samples. Therefore, the results of this study confirm the potential use of ATPS as an appropriate technique to purify bioactive compounds from plant extracts. With the ease of preparation, scaling up ATPS from the laboratory scale is crucial for industrial exploitation. However, further studies need to be carried out to potentially reuse inorganic salts.

## Figures and Tables

**Table 1:** Application of EtOH/inorganic salt in aqueous two-phase systems.

Phase System	Plant Materials	Purified Compounds	References
EtOH/K_3_PO_4_	Saffron stigmas (*Crocus sativus*)	Crocins	[15]
EtOH/(NH_4_)SO_4_	Fruits of Schisandra chinensis Baill	Lignan	[11]
2-propanol/K_3_PO_4_ MeOH/K_2_HPO_4_ (KH_2_PO_4_)	Coffee bean Guarana seed	Caffeine	[16]
EtOH/NaH_2_PO_4_ EtOH/Na_2_S_2_O_3_ EtOH/Na_2_SO_4_ EtOH/Na_2_CO_3_	Chili	Capsaicin	[17]
EtOH/(NH_4_)_2_SO_4_ EtOH/NaH_2_PO_4_	Nitraria tangutorun Bobr Lycium ruthenicum Murr.	Anthocyanins	[18]
Hexafluoroisopropanol/NaCl	Ramie leaves	Chlorogenic acid	[19]
Polyethylen glycol/Na Citrate Acetone/Na citrate	Roselle (*Hibiscus sabdariffa*) calyces	Phenolic compounds	[14]
EtOH/(NH_4_)SO_4_ EtOH/NaH_2_PO_4_ 1-propanol/glucose 1-propanol/mantose	Haskap leaves	Bioactive compounds	[7,20]

**Table 2:** Volume ratio, partition coefficient, and purification efficiency of coffee extract using different aqueous two-phase systems.

Type of ATPS	Volume Ratio (*R*)	Partition Coefficient (*K*)	Purification Efficiency (*E*, %)
ATPS-A	0.33 ± 0.02 ^a^	22.10 ± 1.54 ^a^	88.01 ± 1.42 ^a^
ATPS-B	0.47 ± 0.08 ^b^	17.82 ± 1.99 ^b^	89.19 ± 0.63 ^a^
ATPS-C	0.44 ± 0.02 ^b^	21.08 ± 0.34 ^a^	90.35 ± 0.47 ^a^

Superscript letters in the same column show the significant difference (*p* < 0.05).

**Table 3:** Total polyphenol content (TPC), antioxidant activity (AA), chlorogenic acid, and caffeic acid of coffee extract before and after purification using different aqueous two-phase systems.

APTS EtOH/(NH_4_)_2_SO_4_	TPC (mg GAE/L)	AA (mg TE/L)	Chlorogenic Acid (mmol/L)	Caffeic Acid (mmol/L)
Before purification	37.54 ± 0.96 ^a^	584.50 ± 22.24 ^a^	138.59 ± 7.77 ^a^	218.66 ± 19.94 ^a^
A	38.70 ± 0.84 ^a^	778.05 ± 21.11 ^b^	149.63 ± 12.78 ^a^	233.13 ± 24.80 ^a^
B	38.65 ± 0.96 ^a^	775.39 ± 32.49 ^b^	140.68 ± 19.43 ^a^	209.74 ± 43.16 ^a^
C	38.60 ± 0.83 ^a^	774.06 ± 21.11 ^b^	149.15 ± 19.66 ^a^	218.16 ± 31.97 ^a^

Superscript letters in the same column show the significant difference (*p* < 0.05).

**Table 4:** Composition of aqueous two-phase systems used to purify polyphenols in coffee-pulp extract.

ATPS	(NH_4_)_2_SO_4_ (%)	EtOH (%)	(NH_4_)_2_SO_4_ (g)	EtOH (mL)	H_2_O (mL)	Coffee Pulp Crude Extract (mL)	Top Phase pH	Bottom Phase pH
ATPS-A	18	26	3.6	1.6	9.8	4.8	6.09	5.26
ATPS-B	16	32	3.2	1.9	9.0	5.7	5.92	5.21
ATPS-C	20	28	4.0	1.7	8.8	5.2	6.02	5.26

## Data Availability

Data regarding this article will be provided upon request.
